# Case Report: Case of severe allergic reaction in a child caused by chlorhexidine skin disinfectant

**DOI:** 10.3389/falgy.2026.1812841

**Published:** 2026-05-04

**Authors:** Yujing Zhao, Yingqian Zhang, Juncong Kang, Jingli Zhang, Chenchu Duan

**Affiliations:** Third Department of Respiratory, Hebei Children's Hospital, Key Laboratory of Pediatric Allergy and Immunology of Hebei Province, Innovation Capacity Improvement Project of Hebei Provincial Pediatric Health and Clinical Research Centre, Shijiazhuang, China

**Keywords:** child, chlorhexidine, severe allergic reaction, skin disinfectant, venipuncture

## Abstract

Chlorhexidine is extensively utilized as a skin antiseptic in routine clinical practice. In recent years, reports of life-threatening hypersensitivity reactions induced by chlorhexidine have been increasing progressively. Nevertheless, severe allergic events attributable to chlorhexidine-containing skin disinfectants are frequently misdiagnosed in clinical settings. Herein, we present a pediatric case with recurrent severe allergic reactions following exposure to chlorhexidine-based skin disinfection. This report aims to raise clinicians' awareness of chlorhexidine hypersensitivity.

## Introduction

Disinfectants are common items in daily life. The commonly used topical disinfectants in clinical practice belong to four chemical categories: iodine compounds, chlorine compounds, biguanides and alcohols (1). Chlorhexidine is a synthetic biguanide cationic surfactant with antibacterial, partial antiviral and antifungal activities (2). Owing to its broad antimicrobial activity, it is widely incorporated into medical and healthcare products, as well as cosmetics (3). With the expansion of its application range, reports of chlorhexidine allergy have been on the rise. This article reports a case of severe allergic reaction in a child caused by chlorhexidine skin disinfectant, in order to draw the attention of medical staff.

## Presentation

### Patient information

The patient was a 10-year-old girl. She visited our hospital on March 10, 2025 due to three episodes of severe allergic reactions.

### Clinical findings and timeline

The first episode: She received bromhexine infusion at a local hospital on January 4, 2024. Within 1 min, she experienced swelling and numbness of tongue and lips, generalized red rash, tightness in the throat, shortness of breath, and complained of chest discomfort. Her blood pressure dropped to 93/60 mmHg. The bromhexine infusion was stopped and epinephrine was immediately injected. The respiratory symptoms were relieved in half an hour and the rash subsided several hours later.

The second episode: Next day (January 5, 2024), the patient received intravenous azithromycin for a few seconds and then experienced numbness in her lips, itchy skin rashes, shortness of breath, chest discomfort, vomiting, abdominal pain, and diarrhea. Eventually she lost consciousness. The injection of epinephrine was given again, and the symptoms were relieved after several hours.

The last episode: On November 4, 2024, the patient visited our hospital due to cough and shortness of breath and received intravenous hydrocortisone sodium succinate. About 1–2 min later, she experienced sore throat and drooling, pain in the perianal and perineal areas, generalized red rash, abdominal pain, vomiting, and a sense of impending death. A physical examination revealed wheezing sounds. Her blood pressure dropped to 85/50 mmHg. After intramuscular injections of epinephrine twice, the symptoms gradually improved in 15 min. No biphasic reaction occurred during the 72-hour observation. The details of each allergic reaction episode are presented in Table 1.

**Table 1:** Details of each allergic reaction episode.

Date	Trigger	Interval time	Symptoms	Treatment	Outcome
2024.1.4	Infusion of bromhexine	1 min	Red rashes, swelling and numbness of tongue and lips, tightness in throat, shortness of breath, chest discomfort, blood pressure dropped to 93/60 mmHg	Intramuscular injection of epinephrine	The shortness of breath was relieved in 30 min, and the rash subsided several hours later.
2024.1.5	Infusion of azithromycin	a few seconds	numbness of lips, itchy skin rashes, shortness of breath, chest discomfort, vomiting, abdominal pain and diarrhea; lost consciousness	Intramuscular injection of epinephrine	The symptoms were relieved after several hours.
2024.11.4	Infusion of hydrocortisone sodium succinate	1–2 min	sore throat and drooling, pain in the perianal and perineal areas, red rashes, abdominal pain, vomiting, and a sense of impending death; wheezing, blood pressure dropped to 85/50 mmHg	Administer two intramuscular injections of epinephrine, with an interval of 10 min.	The symptoms were relieved in 15 min. No biphasic reaction occurred during the 72-hour observation.

Medical history: In 2022, she underwent an adenoidectomy at the municipal hospital. The patient experienced no discomfort during intraoperative disinfection with iodophor. However, upon returning to the ward and receiving an intravenous infusion of cefazolin, the patient developed urticaria. After administration of antihistamines, the symptoms were relieved. There was no adverse reaction after taking oral cephalosporins, bromhexine and azithromycin in the past. During hospitalization at our hospital, following application of disinfectant, the skin underlying the ECG monitoring electrode pads developed erythema, papules, and pruritus, with residual hyperpigmentation noted subsequently. There was no history of food allergy, asthma or dermatitis. No family history of allergic diseases.

### Patient perspective

Having experienced three episodes of severe allergic reaction, the patient and her parents were overwhelmed with anxiety. They avoided administering medications during subsequent illnesses and were eager to identify the underlying cause.

### Diagnostic assessment

The patient rapidly developed severe allergic reactions following intravenous infusion of multiple drugs; however, no adverse reactions were documented after prior oral administration of similar medications. What's more, the three pharmaceutical excipients responsible for the severe allergic reactions were distinct from one another; therefore, an allergic reaction induced by the drug substances themselves was ruled out. The patient exhibited no allergic reactions upon exposure to latex gloves during previous surgery; furthermore, latex gloves are not required for venipuncture. So latex allergy is ruled out. Therefore, an allergy to the skin antiseptic is highly suspected in the patient.

The disinfectant used for the patient at the local hospital was 2% chlorhexidine digluconate alcohol skin disinfectant (chlorhexidine 1.8−2.2% W/V, ethanol 63−77% V/V), while the skin disinfectant used in our hospital is a compound preparation (chlorhexidine acetate 0.405−0.495% W/V, povidone-iodine 0.18−0.22% W/V, ethanol 60−70% V/V). The patient had no adverse reactions to povidone-iodine used for skin disinfection during prior surgery, which does not support a diagnosis of povidone-iodine allergy. As both skin antiseptics contain ethanol and chlorhexidine, ethanol may induce skin irritation and is therefore unsuitable for skin prick testing (SPT). Thus, a targeted skin prick test is indicated to confirm whether chlorhexidine is the causative allergen.

We performed a skin prick test (SPT) four months after the last severe allergic reaction. We dropped 75% ethanol on the patient's forearm and observed for 15 min without an allergic reaction. Following skin disinfection with 75% ethanol, a skin prick test was performed on the volar aspect of the patient's forearm using chlorhexidine [5 mg/mL (4)], with negative and positive controls established. The results are shown in Table 2. The diagnosis of chlorhexidine allergy was confirmed.

**Table 2:** Chlorhexidine skin prick test results.

Drug name	Wheal (mm × mm)	Flush (mm × mm)
Chlorhexidine (5 mg/mL)	11 × 12	30 × 30
Histamine positive control (5 mg/mL)	4 × 4	20 × 25
Saline negative control	0 × 0	0 × 0

### Therapeutic interventions

The patient and her parents were counseled to disclose her chlorhexidine allergy to medical staff, review the ingredients of all skin and mucosal antiseptics before use, and avoid all chlorhexidine-containing products.

### Follow-up and outcomes

This patient suffered an arm injury after falling off a bicycle in July 2025; disinfection with benzalkonium chloride was performed with no adverse allergic reactions. There have been no recurrences of severe allergic reaction up to the present.

## Discussion

Chlorhexidine has excellent disinfectant and antiseptic properties, and is therefore widely used in clinical settings in various forms. These include skin disinfectants, mouthwashes, eye drops, medical lubricating gels, dressings, scrubbing solutions, central venous catheters, etc. Among the currently available chlorhexidine skin disinfectants on the Chinese market, most are compound preparations. Commonly used compound components include iodine, ethanol, quaternary ammonium salts, metronidazole, and traditional Chinese medicine ingredients (5). The chlorhexidine-povidone-iodine-ethanol compound disinfectant used for the patient prior to intravenous procedures is widely applied in clinical settings.

Chlorhexidine can be exposed through the skin, mucous membranes and non-intestinal routes, causing type I and type IV allergic reactions. Type IV allergic reactions are mainly manifested as contact dermatitis. In type I allergic reactions, patients may present with local skin symptoms, severe allergic reactions, and even death. There have also been reports that type I and type IV allergic reactions can occur in the same patient (6). In this case, the patient developed erythema, papules and pruritus at the site where the electrode patches were applied after local application of a chlorhexidine-containing skin disinfectant, and there was also pigmentation left behind, suggesting contact dermatitis. A retrospective analysis found that local allergic reactions may occur before severe allergic reactions (7). A French study also showed that 52.9% of patients had experienced mild allergic reactions upon contact with chlorhexidine (8). The patient had a history of local hives and rashes when receiving “cefazolin” infusion, and it is not ruled out that it was a mild allergic reaction after exposure to chlorhexidine.

The exact incidence rate of chlorhexidine allergy is unknown. A study reported a 2% sensitization rate of contact dermatitis induced by chlorhexidine (9). Research on chlorhexidine allergy mainly focuses on the perioperative period. In the United Kingdom, Denmark, and Belgium, chlorhexidine allergy accounts for 9% to 10% of perioperative allergic reactions (10), and 80% of patients present with severe life-threatening allergic reactions of grade 3 to 4 (11). Chlorhexidine has also been confirmed as a common allergen causing perioperative allergic reactions in children (12). During surgery, the situation of contact with chlorhexidine through at least one route was as high as 73.5% (13), and the most common ones were urinary catheter lubricants, chlorhexidine-coated central venous catheters, and local chlorhexidine solutions (14). Therefore, guidelines in countries such as New Zealand and the United Kingdom recommend that all patients suspected of perioperative allergic reactions undergo routine chlorhexidine allergy-related tests (15, 16).

Severe allergic reactions caused by chlorhexidine are rare in children. In 1984, Japan reported the first case of an 9-year-old child experiencing anaphylactic shock due to chlorhexidine allergy (17). France has summarized 17 cases of children allergic to chlorhexidine, among which 13 children experienced severe allergic reactions of grade 2 to 4, and 41.2% of the children required hospitalization. Severe allergic reactions to chlorhexidine in children usually occur at home, and the common cause is the disinfection of broken skin with skin disinfectants (8, 18, 19). Chlorhexidine is well tolerated on the healthy skin of children. However, in damaged skin, the absorption of chlorhexidine can increase by 100 times (20), so even a trace amount of chlorhexidine can cause severe allergic reactions. In this case, the pediatric patient experienced multiple severe allergic reactions that occurred rapidly within minutes after intravenous infusion in the hospital, which is considered to be related to the intravenous puncture. Xiao Hao summarized the cases of confirmed chlorhexidine allergy in China. Among the 30 allergic reactions that occurred, 22 were due to skin disinfection before venipuncture. This may be because chlorhexidine is widely used as a skin disinfectant before venous procedures in our country. Residual chlorhexidine components on the skin or incomplete drying of the disinfectant before venipuncture can both lead to trace amounts of chlorhexidine entering the bloodstream (21). A survey on the use of skin disinfectants for newborns in China shows that only 55.6% of the time, the skin is dried after each use of chlorhexidine-containing skin disinfectant solution (22).

Because chlorhexidine allergy is difficult to identify, it is often confused with drug allergy in clinical practice, especially with antibiotics. This leads to excessive avoidance of drugs and increases the risk of severe allergic reactions for patients upon subsequent exposure. The patient had experienced three severe allergic reactions before the diagnosis was confirmed. Similar cases reported in China also showed that patients had suffered multiple severe allergic reactions before the diagnosis was made (23, 24). This requires the attention of all medical staff. Japan, the United States, the United Kingdom, Australia, New Zealand, Canada, France, and other countries have all issued warnings regarding chlorhexidine, emphasizing the need to establish strict usage rules and raise awareness of the related allergic risks (8). Since 2017, the US Food and Drug Administration has required manufacturers to mention the risk of severe allergic reactions when labeling over-the-counter drugs containing chlorhexidine (25).

Frequent exposure and the use of high-concentration chlorhexidine can increase the risk of sensitization (26). Some researchers believe that the use of chlorhexidine disinfectant for umbilical cord care in the neonatal period is the cause of early percutaneous sensitization in some children (1). The 2021 “Guidelines for Neonatal Skin Management in Intensive Care Units” in China emphasizes that it is not recommended to routinely use disinfectants to prevent infection at the umbilical stump of newborns, and it does not recommend using chlorhexidine to disinfect the skin (27). The “Technical Specifications for Venous Therapy Nursing Operations” in 2013 suggested that chlorhexidine should be used with caution for infants under 2 months of age (28), but this was not mentioned in the 2023 technical operation specifications (29). Currently, the concentrations of chlorhexidine used in various countries vary significantly. According to the current “General Requirements for Skin Disinfectants” in China, the recommended content of guanidine active ingredients is 2 g/L to 45 g/L. In Denmark, 0.5% chlorhexidine is used for skin disinfection before all procedures. In the UK, it is recommended to use 2% chlorhexidine for skin disinfection before venipuncture (30). In Japan, it is suggested to use a minimum concentration of 0.05% chlorhexidine on the wound surface, and its application on mucous membranes is prohibited (31). Nishihara's research compared the efficacy of 0.5%, 1%, and 2% chlorhexidine ethanol solutions for local skin disinfection. The results showed no significant difference in the antibacterial efficacy among the three concentrations (32). Much research is still needed in the future to determine the minimum effective concentration of chlorhexidine for skin disinfection, in order to balance the risk of infection and the risk of sensitization.

There are many deficiencies in the diagnosis of this case. Serum tryptase was not measured during any acute episode due to technical limitations. Patch testing for delayed-type chlorhexidine allergy was not available at our centre and was therefore not performed. For chlorhexidine immediate-type allergy, the combination of SPT and serum sIgE detection has the highest sensitivity and specificity for diagnosis (33). There are no commercially available chlorhexidine-specific IgE serum detection reagents in China, so only the skin prick test was performed in this case. However, the sensitivity of SPT in evaluating chlorhexidine allergy reactions is 95%, and the specificity is 97% (33). Secondly, for patients allergic to chlorhexidine, alternative disinfectants should be sought. For instance, a hospital in the UK uses 70% isopropyl alcohol wipes for skin disinfection before peripheral venous catheterization and povidone-iodine alcohol solution for preoperative skin disinfection (34). Due to the few reports of allergic reactions caused by povidone-iodine in children (35), it is recommended to conduct a povidone-iodine skin test to determine if it can be used as an alternative disinfectant.

## Conclusion

Chlorhexidine is widely used in clinical settings. Herein, we report a pediatric case of recurrent severe anaphylactic reactions induced by chlorhexidine-based skin disinfectants. This case report aims to heighten clinicians' awareness of chlorhexidine hypersensitivity. Rational application, standardized operation and accurate identification of medical products containing chlorhexidine should be carried out to minimize accidental exposure and reduce the incidence of severe allergic reactions.

## Data Availability

The original contributions presented in the study are included in the article/Supplementary Material, further inquiries can be directed to the corresponding author.
